# Age-related downregulation of dihydrotestosterone-inactivating enzymes in human scalp sebaceous glands

**DOI:** 10.1186/s41232-026-00417-5

**Published:** 2026-04-07

**Authors:** Yasuaki Ikuno, Yukie Kande, Akiko Arakawa, Akihiko Yamaguchi, Toshifumi Takahashi, Noriki Fujimoto, Hayato Naka-Kaneda

**Affiliations:** 1 Department of Dermatology, Shiga University of Medical Science, Otsu, 520-2192 Japan; 2 Department of Anatomy, Shiga University of Medical Science, Otsu, 520-2192 Japan; 3 Department of Dermatology, Ludwig-Maximilian-University, Munich, 80337 Germany; 4 Department of Dermatology, Leipzig University, Leipzig, 04109 Germany

**Keywords:** AKR1C, Sebaceous glands, Aging, Hair follicle

## Abstract

**Background:**

Aging alters the systemic steroid environment, including reductions in circulating androgens. In peripheral tissues, androgen exposure is regulated mainly by local steroid metabolism, which can activate or inactivate androgens independently of systemic concentrations. In the scalp, 5α-reductase–mediated generation of dihydrotestosterone (DHT) has been studied extensively in relation to conditions such as androgenetic alopecia. In contrast, age-related regulation of DHT-inactivating pathways, including the aldo–keto reductase family 1 member C (AKR1C) family, remains insufficiently defined.

**Methods:**

We examined AKR1C1–4 expression in scalp tissues from individuals of different ages, sexes, and hair loss conditions using immunohistochemistry (IHC), quantitative PCR (qPCR), and reanalysis of publicly available RNA-sequencing (RNA-seq) datasets. Androgen receptor (AR) localization was assessed in multiple scalp compartments. *AKR1C* expression was also analyzed in immortalized human sebaceous gland cells (SEB-1) and dermal papilla cells (DPCs). In addition, the effect of sulforaphane on AKR1C expression was evaluated.

**Results:**

*AKR1Cs* were strongly expressed in male sebaceous glands (SGs) but declined with age. AKR1C4, previously considered liver specific, was detected in SGs and hair follicles (HFs) by IHC and further confirmed by qPCR in SEB-1 cells and DPCs. AR expression was predominant in SGs, with lower levels in sweat glands, the epidermal granular layer, and the HF infundibulum, but not detected in dermal papillae and bulge regions. AR localization was cytoplasmic in the epidermis but nuclear in SGs and sweat glands, and was unaffected by age, sex, or hair loss condition. Sulforaphane treatment upregulated *AKR1C* expression in both SEB-1 cells and DPCs.

**Conclusions:**

We identify scalp SGs as a principal site of AR expression and AKR1C-mediated androgen inactivation, including AKR1C4, and demonstrate that AKR1C expression in SGs is sex dependent and declines with age. These findings suggest that age-associated changes in SG steroid metabolism may influence local androgen metabolism within the scalp microenvironment, with implications for HF homeostasis during aging.

**Supplementary Information:**

The online version contains supplementary material available at 10.1186/s41232-026-00417-5.

## Background

Hair follicle (HF) behavior in adult skin is strongly influenced by local androgen availability, and the regulation of this microenvironment depends not only on circulating hormones but also on steroid metabolism within peripheral tissues [1, 2]. The systemic steroid environment changes with aging, with circulating androgens declining and sex hormone-binding globulin increasing [3]. In contrast, end-organ androgen exposure is determined primarily by intracrine enzyme systems that generate, activate, or inactivate androgens within tissues [1]. In the skin, 5α-reductases (5αRs), including steroid 5 alpha-reductase 1–3 (SRD5A1-3), convert testosterone into dihydrotestosterone (DHT), the more potent receptor-active androgen, and the aldo–keto reductase family 1 member C1–4 (AKR1C1–4) exhibit 3α-hydroxysteroid dehydrogenase (3α-HSD) activity and convert DHT into less active metabolites such as 3α-androstanediol [4]. These enzymatic pathways are highly tissue specific, and local androgen action is therefore regulated within individual tissues in a manner that is largely independent of serum hormone levels [2].

In the regulation of skin appendages, androgen-dependent processes are also important in the human scalp, as microenvironmental androgen activity influences HF cycling and the functional properties of associated appendages [5]. The sebaceous gland, an androgen-responsive structure closely associated with the HF, contains a diverse set of steroid-metabolizing enzymes, suggesting that it contributes to the control of the local DHT milieu [6]. Although androgen-activating pathways such as 5α-reduction have been studied extensively, information regarding age-related changes in DHT-inactivating mechanisms within the human scalp remains limited.

Androgenetic alopecia (AGA), a common age-associated and androgen-dependent condition, has been linked to excessive DHT signaling within the HF microenvironment [7]. Epidemiological studies show that by around the age of 50, approximately 50–80% of men and 40–50% of women exhibit visible signs of AGA [8]. Clinical and histological findings indicate that DHT-related processes contribute to follicular miniaturization and alterations in dermal papilla cell (DPC) function, including the upregulation of a 5αR member, SRD5A2, increased androgen receptor (AR) expression, impaired growth factor production, and senescence-associated changes [9–18]. These observations suggest that enhanced androgen signaling affects both the niche environment and the intrinsic properties of HF cells.

Despite the substantial attention given to DHT synthesis in AGA-related research, pathways responsible for DHT inactivation, such as AKR1Cs, remain insufficiently characterized. In particular, their expression patterns in the human scalp and their regulation with aging have not been systematically examined. Therefore, in this study, we investigated the expression patterns of AKR1Cs in the human scalp across different ages and sexes, to clarify their potential relevance to age-associated changes in local androgen metabolism.

## Methods

### Transcriptome data analysis

**Fig. 1:**
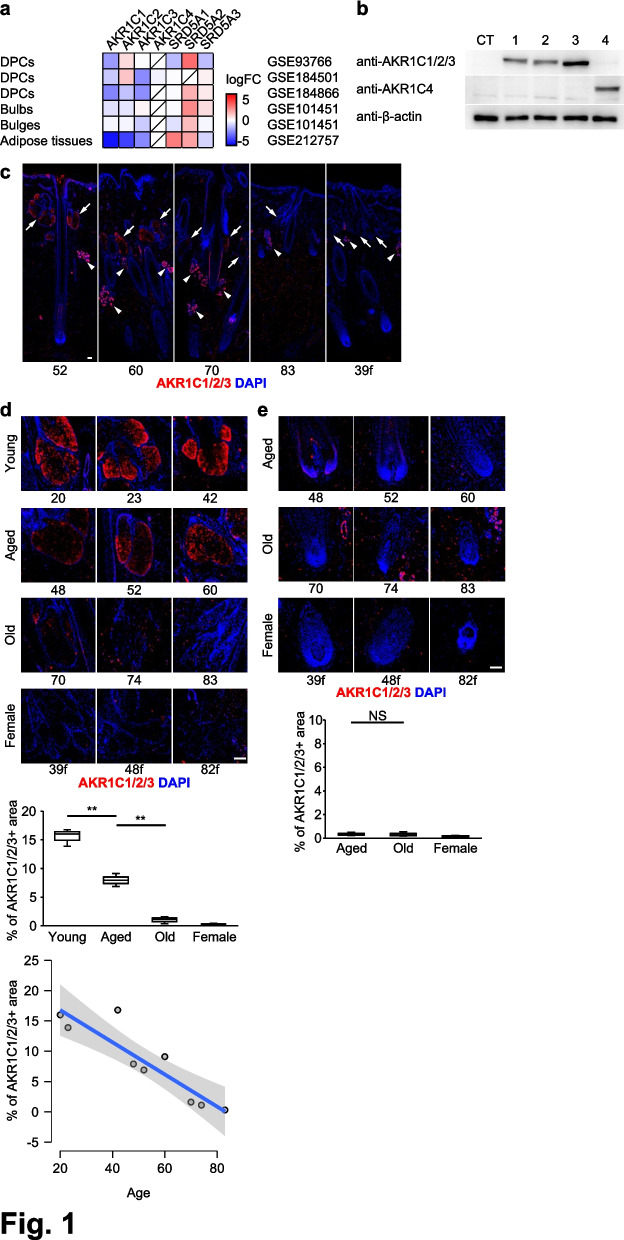
AKR1C1/2/3 expression declines in male SGs with age. **a** Heatmap of RNA-seq data showing downregulation of *AKR1Cs* and upregulation of *SRD5A2* in DPCs, bulbs, bulges, and adipose tissues in AGA scalps. **b** Western blotting of mKate2-tagged AKR1C1–4 (lanes 1–4) and mKate2 alone (lane CT) shows that the anti-AKR1C1 antibody (GeneTex, GTX105620) recognizes AKR1C1/2/3, whereas the anti-AKR1C4 antibody (Atlas Antibodies, HPA044720) is specific to AKR1C4. (**c**) IHC shows AKR1C1/2/3 expression in SGs, sweat glands, and scattered dermal cells (arrows, SGs; arrowheads, sweat glands). **d** High magnification images show strong AKR1C1/2/3 expression in male SGs that decreases with age, with undetectable expression in female SGs. Age groups for display: Young 20–42 y, Aged 48–60 y, Old 70–83 y (*n* = 3 each). A simple linear regression line (β = − 0.266, R^2^ = 0.82) is shown in the scatter plot to illustrate the relationship between AKR1C1/2/3 positive area and age. **e** No AKR1C1/2/3 expression was detected in HFs of either sex. Numbers below each image indicate the age (years) of the individual donor; “f” denotes female. Box plots show the median, quartiles, and range of the data. Scale bars, 100 μm

Publicly available RNA-seq datasets were reanalyzed using GREIN (GEO RNA-seq Experiments Interactive Navigator; http://www.ilincs.org/apps/grein/), and the results are shown in Fig. 1a and Fig. 4a. Gene-level raw counts (read counts) were obtained from the “Counts table”, and log2 fold change (logFC) values were retrieved from the “Signature table” generated by the GREIN platform. Sample provenance (tissue/cell type), species, sample source/condition, age (when available) and sex are summarized in Supplementary Table S1.

### Human scalp tissues

Histologically normal scalp tissues were obtained from 12 patients (9 males and 3 females; aged 20–83 years) with clear clinical records who underwent surgery at the Department of Dermatology, Shiga University of Medical Science, from 2010 to 2024. Samples were taken from the parietal, frontal, temporal, and occipital scalp regions (Table 1). For age-stratified analyses, male samples were categorized into three groups based on donor age: Young (20–42 years), Aged (48–60 years), and Old (70–83 years).

**Table 1:** Information on human scalp tissue samples

Patient no	Age (year)	Gender	Sampling site	Past history	Medications	Hamilton–Norwood scale	Ludwig scale	Biopsy site hair status
1	20	M	Parietal	None	None	I		Hairy
2	23	M	Parietal	None	None	III		Thinning
3	42	M	Frontal	Disc herniation	Pregabalin	III		Thinning
4	48	M	Temporal	Gastric ulcer	Ascorbic acid, calcium pantothenate	II		Hairy
5	52	M	Temporal	Ureterolithiasis	None	III		Hairy
6	60	M	Occipital	None	None	III		Hairy
7	70	M	Occipital	Diabetes, prostate cancer, hypertension	Metformin, aspirin, telmisartan, teneligliptin, carbocisteine, gliclazide	III		Hairy
8	74	M	Parietal	Hypertension	Amlodipine	IV		Hairy
9	83	M	Parietal	Alcoholic liver disease, dyslipidemia, hyperuricemia, palmoplantar pustulosis	Biotin, ambroxol, dextromethorphan, bezafibrate, famotidine, allopurinol, sennoside	VII		Thinning
10	39	F	Occipital	Hypothyroidism	None		I-1	Hairy
11	48	F	Parietal	None	Ascorbic acid		I-1	Hairy
12	82	F	Parietal	Hypertension, dimentia, cerebral infarction, dyslipidemia	Quetiapine, aspirin, clopidogrel, fluvastatin, lansoprazole		I-2	Hairy

Abbreviations: M, male; F, female

Age = the age (year) of sampling. Sampling site = the site where the specimen was collected

### Immunohistochemistry (IHC)

Tissue samples were fixed overnight at 4 °C in 10% neutral-buffered formalin and embedded in paraffin. Paraffin blocks were sectioned at 4 μm, deparaffinized, and subjected to heat-induced antigen retrieval in Tris-ethylenediaminetetraacetic acid buffer (pH 9.0) at 121 °C for 20 min. Tissue sections were stained with antibodies (Supplementary Table S2). Imaging and quantification were performed using an IX83 microscope (Olympus) and ImageJ/Fiji software (National Institutes of Health).

### Cell culture

SEB-1 male-derived immortalized sebocyte cells were kindly provided by the Penn State Research Foundation and cultured in Reduced Serum DMEM/Ham's F-12 Medium (Nacalai Tesque) supplemented with 5% fetal bovine serum, 180 μM adenine, 0.4 μg/ml hydrocortisone, 10 ng/ml epidermal growth factor, and 10 μM isoproterenol. Human DPCs, derived from the temple region of a 53-year-old Caucasian male, were obtained (c-12071, PromoCell) and cultured in Follicle Dermal Papilla Cell Growth Medium (Ready-to-use) (c-26501, PromoCell). All cells were maintained in a humidified incubator at 37 °C under 5% CO_2_.

SEB-1 cells and DPCs were cultured with or without 10 µM sulforaphane (SFN; 10,496, Cayman Chemical), an *NFE2 like bZIP transcription factor 2* (*NFE2L2,* also known as *NRF2*) activator, 100 nM DHT (A0462, Tokyo Chemical Industry) or dimethyl sulfoxide (13,407–45, Nacalai Tesque) as a vehicle control for 48 h.

### Immunocytochemistry (ICC)

Cells cultured in 48-well plates were fixed with 4% paraformaldehyde for 15 min at room temperature and subjected to antigen retrieval using HistoVT One (06380–05, Nacalai Tesque) according to the manufacturer’s instructions. After permeabilization and blocking, cells were stained with antibodies (Supplementary Table S2). Nuclei were counterstained with DAPI, and images were acquired using an IX83 microscope (Olympus).

### Alkaline phosphatase staining

Alkaline phosphatase (ALP) activity was assessed using a chromogenic method based on nitro blue tetrazolium (NBT) and 5-bromo-4-chloro-3-indolyl phosphate (BCIP). Cells cultured in 24-well plates were fixed with 4% paraformaldehyde for 15 min, followed by phosphate-buffered saline (PBS) washes, permeabilized with ice-cold acetone, ethanol, 1:1, for 2 min, and washed again with PBS. Cells were then incubated with BCIP-NBT Solution, Ready To Use (19,880–84, Nacalai Tesque) at 37 °C for 10–20 min. ALP-positive cells were identified by dark blue to purple precipitates under brightfield microscopy, and images were acquired using an IX83 microscope (Olympus).

### Western blot analysis

Cell lysates were prepared in radioimmunoprecipitation assay buffer (Nacalai Tesque), and western blotting was performed using standard methods, with a polyacrylamide gel electrophoresis and blotting system (Atto). Imaging and quantification were performed using a FUSION SOLO.6S.EDGE system (Vilber), and ImageJ software (National Institutes of Health).

### qPCR analysis

Total RNA was extracted using NucleoZOL (Macherey–Nagel) and reverse-transcribed into cDNA using ReverTra Ace (Toyobo). Quantitative PCR (qPCR) was performed with THUNDERBIRD Next SYBR qPCR Mix (Toyobo) on a StepOnePlus Real-Time PCR System (Applied Biosystems). The primer sequences used in this study are listed in Supplementary Table S3.

### Statistical analysis

Data from three or more independent experiments were used for statistical analyses. Statistical significance was determined using two-tailed unpaired Welch’s *t*-tests. *P* < 0.05 was considered statistically significant. *P < 0.05, **P < 0.01, ***P < 0.001.

To evaluate whether age, scalp site, local involvement, sex, or Hamilton–Norwood (HN) severity independently affected AKR1C1–4 expression, we fit separate ordinary least-squares (OLS) linear regressions for each gene with expression level as the dependent variable. Age (years, continuous) and site indicators were included as predictors, with occipital scalp coded as the reference category. Site categories without observations in a given subset (e.g., frontal) were excluded to avoid singularity. Local involvement was coded as 0 = Hairy and 1 = Thinning. Sex was coded as 0 = female and 1 = male, and HN severity was coded as I–III = 1, IV–V = 2, VI–VII = 3 [19].

Given the limited sample size, we prespecified a minimal primary model to prioritize estimation stability and avoid overfitting: age + site (occipital reference) + local involvement. Because HN severity is ordinal, we examined its association with age using Spearman’s rank correlation (HN coded I–III = 1, IV–V = 2, VI–VII = 3) and observed a strong positive correlation (ρ = 0.77, P = 0.015). Given this collinearity and the reduction in residual degrees of freedom, HN severity was excluded from the primary model and evaluated only in prespecified sensitivity analyses. Likewise, sex was examined only in sensitivity analyses.

To further assess robustness with respect to scalp-site confounding, we conducted a prespecified parietal-only sensitivity analysis (site held constant), in which age was the sole predictor. With n = 9, the minimal model retained three to four residual degrees of freedom (df = 3–4).

Analyses were performed in Microsoft Excel (OLS). We report unstandardized coefficients (β) with standard error (SE), t value, two-tailed p value, and 95% confidence interval (CI). Model fit was summarized with R^2^, adjusted R^2^, and the F statistic. To quantify the independent contribution of age across models, partial R^2^ for age was also reported. Given the small residual degrees of freedom, inference emphasizes effect-size estimation (β, 95% CIs, partial R^2^) and prespecified sensitivity analyses rather than dichotomous significance testing. For visualization, simple regression lines were drawn for illustration; all inferential conclusions are based on the multivariable models (Supplementary Tables S4–S6).

## Results

### AKR1C expression is downregulated in AGA scalps

We first examined the expression profiles of *AKR1Cs* and *SRD5As* in AGA scalps using publicly available RNA-seq datasets by GREIN. As expected, *SRD5A2*, the main target of the representative 5αR inhibitor finasteride, was upregulated in AGA scalps (Fig. 1a). Notably, we also confirmed downregulation of *AKR1Cs* in DPCs, bulbs, bulges, and adipose tissues in AGA scalps. Quantitatively, *AKR1C1/2* transcripts (read counts) were considerably more abundant than *SRD5A2*, suggesting the potential significance of *AKR1C* downregulation in androgen excess (Table 2).

**Table 2:** Average RNA-seq read counts for each gene

	***AKR1C1***		***AKR1C2***		***AKR1C3***		***AKR1C4***		***SRD5A1***		***SRD5A2***		***SRD5A3***		**GSE accession**
	Hairly	Bald	Hairly	Bald	Hairly	Bald	Hairly	Bald	Hairly	Bald	Hairly	Bald	Hairly	Bald	
DPCs	78.60	20.72	6.89	10.98	0.61	0.62	0.10	0.00	15.32	6.29	0.22	1.62	87.80	37.05	GSE93766
DPCs	62.52	30.99	10.51	22.96	5.52	1.17	0.10	0.08	6.46	6.59	0.00	0.23	15.81	18.81	GSE184501
DPCs	516.38	99.61	15.83	7.18	20.86	4.56	0.00	0.00	20.15	19.65	0.21	0.61	14.34	18.80	GSE184866
Bulbs	2.00	1.39	1.16	0.85	1.00	1.12	0.00	0.00	28.62	28.03	0.06	0.27	2.77	4.30	GSE101451
Bulges	3.12	2.84	3.17	2.89	8.40	4.31	0.00	0.11	156.71	107.57	0.15	0.37	10.16	9.41	GSE101451
Adipose tissues	822.36	33.54	444.77	33.54	105.77	21.56	0.00	0.00	61.67	358.60	0.69	2.40	20.67	11.98	GSE212757

### AKR1C1/2/3 expression declines in male sebaceous glands with age

To investigate precise AKR1C protein expression, we first validated antibody specificity due to the high sequence homology among AKR1C family members using human embryonic kidney 293 T (HEK293T) cells overexpressing each AKR1C. The anti-AKR1C1 antibody (GeneTex, GTX105620) recognized AKR1C1/2/3 proteins, whereas the anti-AKR1C4 antibody (Atlas antibodies, HPA044720) selectively detected AKR1C4 (Fig. 1b and Supplementary Fig. S1). Using these validated antibodies, we examined age-related changes in AKR1C expression in male and female scalp tissues by immunohistochemistry (IHC) (Table 1). Strong AKR1C1/2/3 immunoreactivity was detected in sebaceous glands (SGs) and sweat glands, and scattered dermal cells (Fig. 1c). Notably, the AKR1C1/2/3-positive (+) area in male SGs progressively decreased with age, whereas the signal was hardly detectable at any age in female SGs. This age-dependent decline was evident when male samples were stratified into Young, Aged, and Old groups. Consistent with this observation, analysis using age as a continuous variable revealed a clear negative correlation between AKR1C1/2/3-positive area and age (Fig. 1d), which remained significant after accounting for potential confounding factors, including AGA severity and sampling site (Supplementary Table S4). Furthermore, AKR1C1/2/3 expression was not detected in HFs of either sex (Fig. 1e).

### AKR1C4 expression declines in sebaceous glands and hair follicles with age

**Fig. 2:**
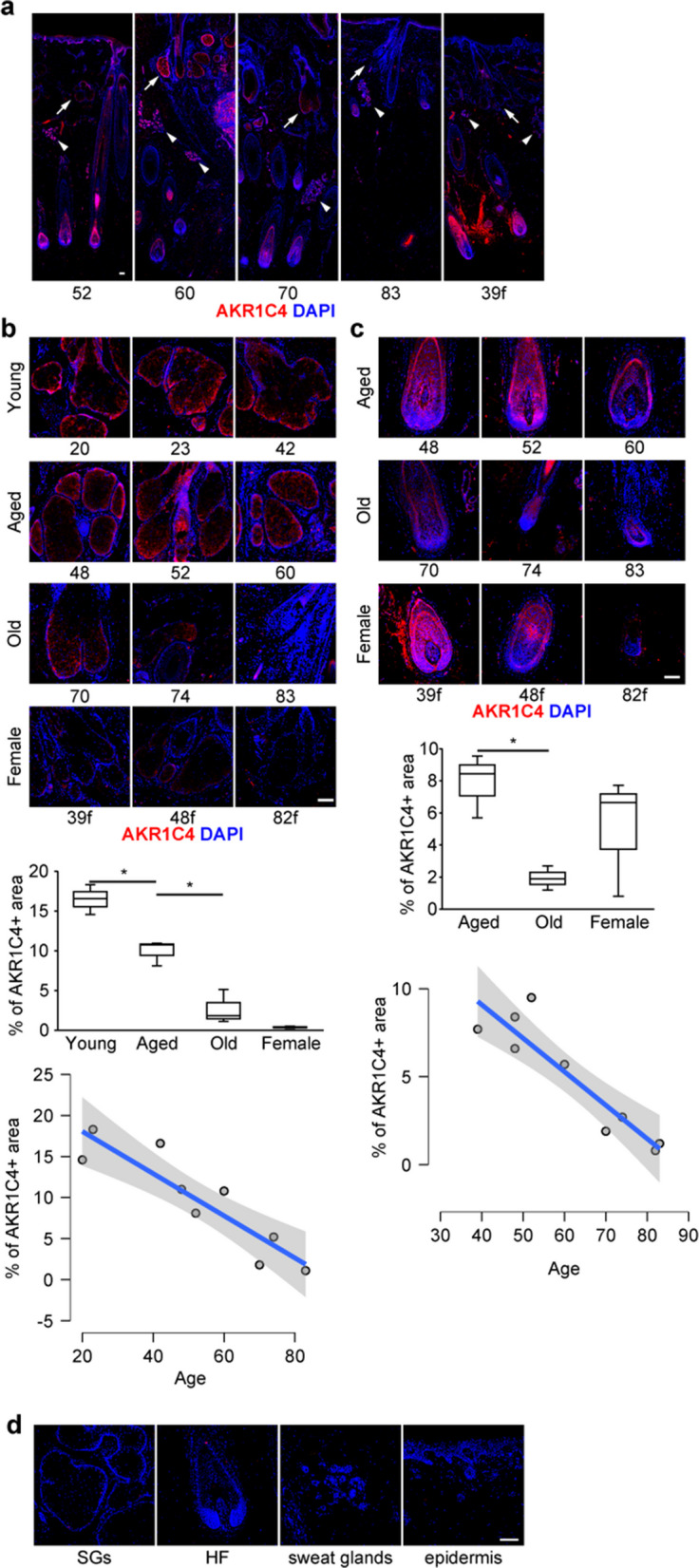
AKR1C4 expression declines in SGs and HFs with age. **a** IHC shows AKR1C4 expression in SGs, sweat glands, and HFs (arrows, SGs; arrowheads, sweat glands). **b** High magnification images show strong AKR1C4 expression in male SGs that decreases with age, with low expression in female SGs. **c** High magnification images of HFs show strong AKR1C4 expression in HFs of both sexes and decreases with age, although the statistical evaluation of AKR1C4 expression in female samples was limited by the sample size. Age groups for display: Young 20–42 y, Aged 48–60 y, Old 70–83 y (n = 3 each). Simple linear regression lines are shown in the scatter plots to illustrate the relationship between AKR1C4 positive area and age (SGs, β = − 0.257, R^2^ = 0.82; HFs, β = − 0.190, R^2^ = 0.84). **d** Isotype control staining yielded no detectable signal. Numbers below each image indicate the age (years) of the individual donor; “f” denotes female. Box plots show the median, quartiles, and range of the data. Scale bars, 100 μm

AKR1C4, previously considered liver specific, showed a similar expression pattern to AKR1C1/2/3 in male SGs (Fig. 2a). In male SGs, the AKR1C4-positive area showed an age-dependent reduction, as demonstrated by both categorical comparisons and its negative correlation with chronological age (Fig. 2b and Supplementary Table S5). AKR1C4 was also expressed in HFs, and its expression declined with age in both sexes, although statistical evaluation in female samples was limited by sample size (Fig. 2c and Supplementary Table S6). To examine whether anatomical location or biopsy-site hair status influenced AKR1C expression, we performed multivariable linear regression analyses adjusting for scalp region and biopsy-site hair status. Across all models, no significant regional effects were observed for AKR1C1–3 in sebaceous glands or for AKR1C4 in sebaceous glands (Supplementary Tables S4 and S5). In contrast, AKR1C4 expression in hair follicles was higher in parietal samples than in occipital samples (Supplementary Table S6). Biopsy-site hair status was not independently associated with AKR1C expression in any model. Isotype control staining yielded no detectable signal, confirming antibody specificity (Fig. 2d). Together, these results suggest that AKR1C4, similar to other AKR1C family members, exhibits age-related downregulation in both SGs and HFs.

### Active AR is expressed primarily in SGs rather than in dermal papillae or bulges

**Fig. 3:**
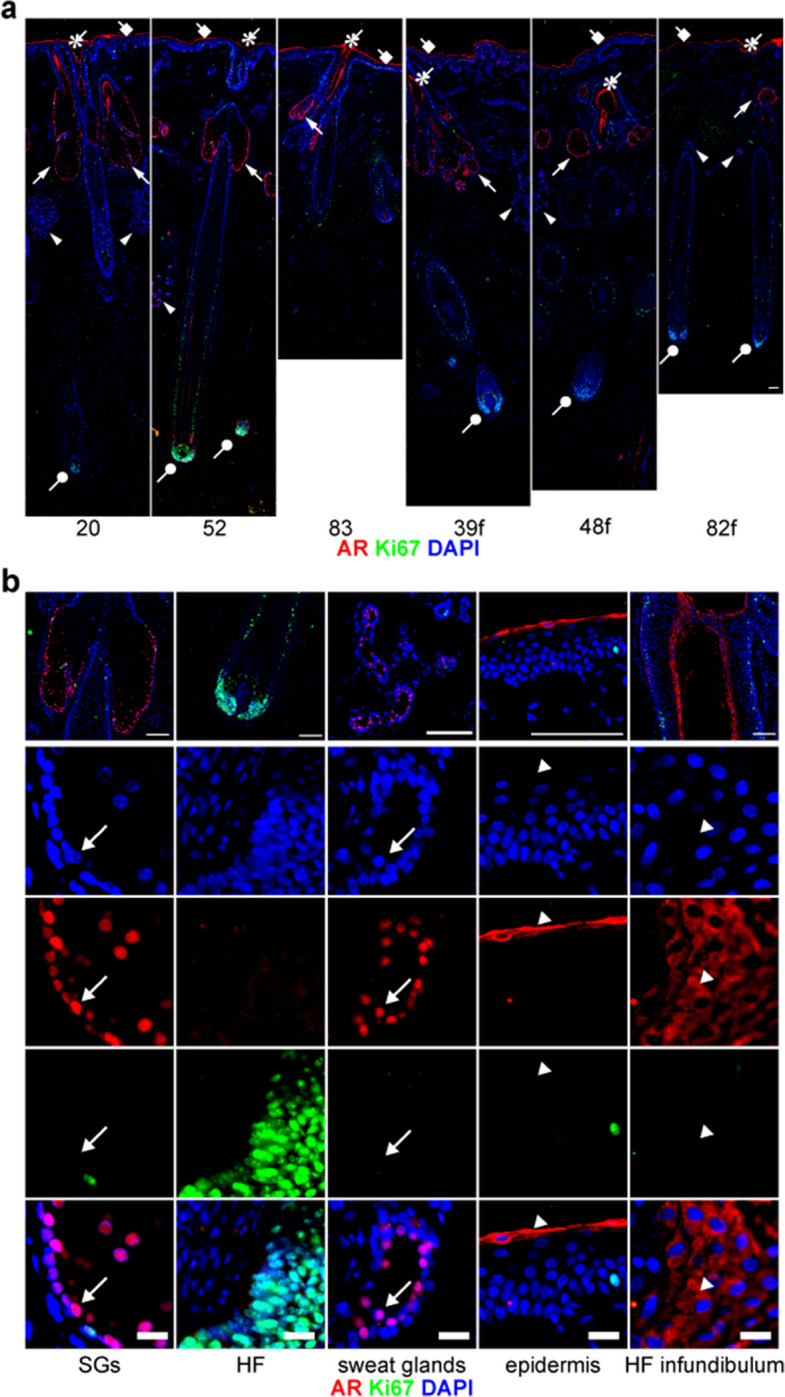
Active AR is expressed primarily in SGs rather than in dermal papillae or bulges. **a** IHC shows AR expression in SGs (arrows), sweat glands (arrowheads), the epidermal granular layer (squares), and the HF infundibulum (asterisks), but not in dermal papillae (circles) or bulge regions. **b** High-magnification images reveal that nuclear-localized (active) AR is predominant in SGs and present at lower levels in sweat glands, whereas AR in the epidermal granular layer and HF infundibulum is localized mainly in the cytoplasm. Lower panels show enlarged views of representative regions with individual AR, Ki67, and DAPI channels and their merged images. Arrows indicate representative nuclear localization of AR, whereas arrowheads indicate cytoplasmic localization of AR. Numbers below each image indicate the age (years) of the individual donor; “f” denotes female. Scale bars, 100 μm (a and upper panels of b) and 20 μm (lower panels of b)

AR is known to be expressed in SGs and DHT-exposed DPCs. However, the age-related changes in AR expression in human scalp tissues have not been systematically investigated. In our IHC analysis with anti-AR antibody (Proteintech, 22,089–1-AP), AR expression was predominant in SGs, with lower levels in sweat glands, the epidermal granular layer, and the HF infundibulum. No AR expression was detected in DPCs or bulge regions (Fig. 3a). The AR expression patterns were unchanged by age, sex, or hair loss conditions. Notably, AR localization differed between tissues, being cytoplasmic in the epidermis but nuclear in SGs and sweat glands (Fig. 3b). To further validate these AR staining patterns, we performed additional IHC using a second AR antibody (AR (CST), Cell Signaling Technology, #5153). This antibody detected nuclear AR in SGs and sweat glands, whereas the cytoplasmic AR signals observed in the epidermis and HF infundibulum with the Proteintech antibody were not detected (Supplementary Fig. S2). This difference may reflect the distinct epitope specificities of the two antibodies, with the Proteintech antibody recognizing an N-terminal region of AR and the CST antibody being raised against the full-length (FL) protein. As the CST antibody did not detect cytoplasmic signals attributable to N-terminal isoforms in our IHC analysis, its reactivity may depend, at least in part, on epitopes located within the C-terminal region. These results suggest that SGs, rather than dermal papillae or bulges, represent the primary androgen-responsive tissue in the human scalp.

### Sulforaphane upregulates AKR1C expression

**Fig. 4:**
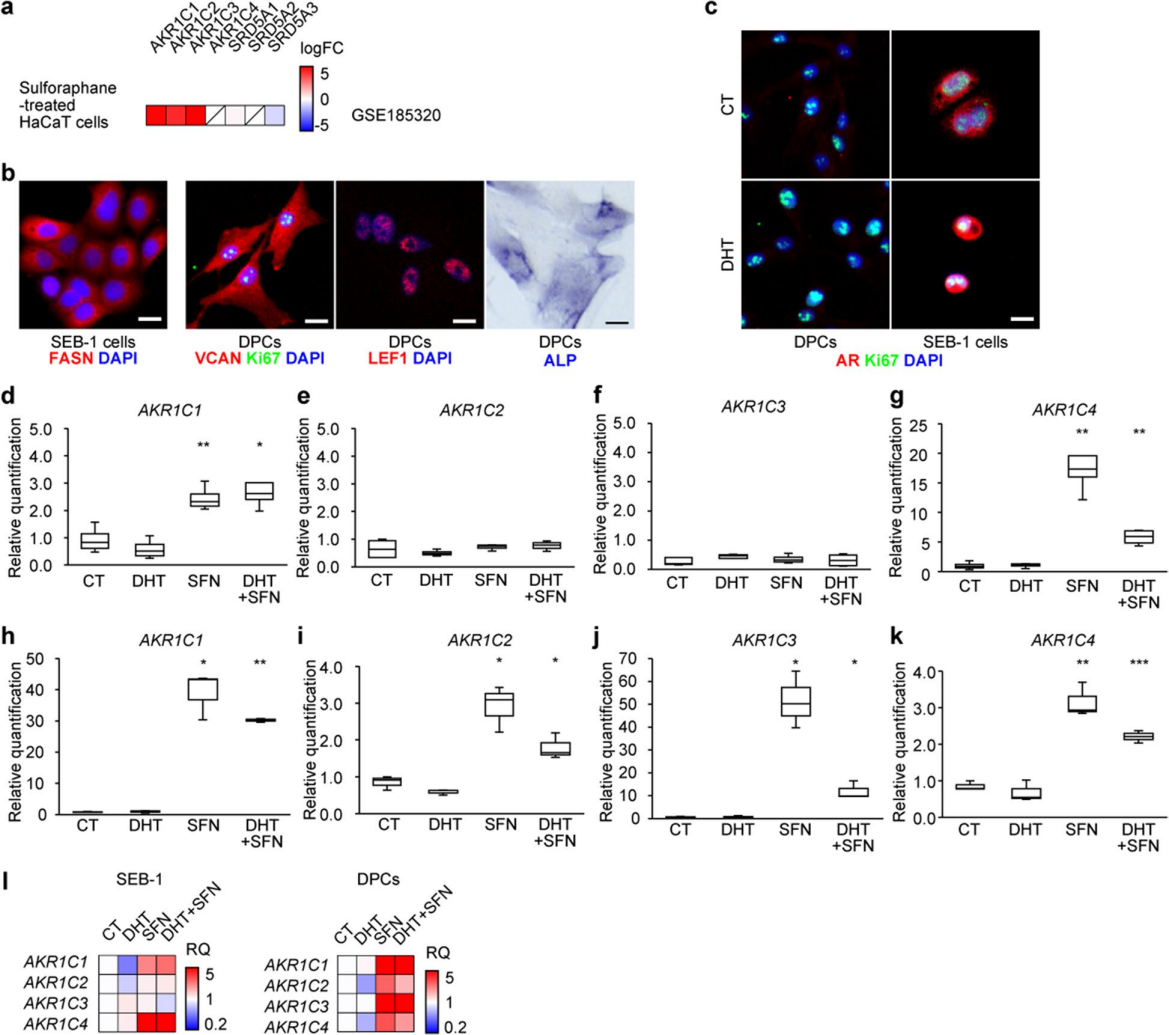
SFN upregulates *AKR1C* expression in SEB-1 cells and human DPCs. **a** Heatmap of RNA-seq data showing upregulation of *AKR1Cs* in sulforaphane (SFN)-treated HaCaT cells. **b** ICC of SEB-1 cells for FASN and of DPCs for VCAN and Ki67. **c** ICC of SEB-1 cells and DPCs for AR and Ki67 under control (CT) and DHT-treated conditions. (d-g) qPCR results showing AKR1C1-4 expression in SEB-1 cells treated with or without SFN and DHT. **h**–**k** qPCR results showing AKR1C1-4 expression in human DPCs treated with or without SFN and DHT. **l** Heatmaps generated from the qPCR data in panels d-k. Box plots show the median, quartiles, and range of the data. Statistical significance in (**d**-**k**) was determined by comparison with the control (CT) group. *P < 0.05, **P < 0.01. Scale bars, 20 μm

5αR inhibitors can cause serious side effects due to systemic DHT suppression [20]. In contrast, AKR1C activation enables local DHT inactivation without inhibiting DHT synthesis. Sulforaphane (SFN), an *NFE2L2* (*NRF2*) activator, induces *NRF2* target genes involved in cellular antioxidant and detoxification responses, including members of the *AKR1C* family, across multiple cell types [21, 22]. SFN has also been shown to promote hair growth in diabetic ob/ob mice, possibly by inducing 3α-HSDs such as *Akr1c21* and *Dhrs9* [23]. To evaluate the effect of SFN on AKR1C expression in the skin, we first reanalyzed public RNA-seq data from human keratinocyte HaCaT cells. Although *AKR1C4* and *SRD5A2* were not included, SFN markedly induced *AKR1C1/2/3* (Fig. 4a). We next examined AKR1C regulation in our in vitro models, which included the male-derived immortalized sebocyte line SEB-1 cells [24] and DPCs. Previous work using the female-derived immortalized sebocyte line SZ95 has suggested that sebocytes may participate in the local regulation of androgen homeostasis in human skin by both synthesizing and inactivating active androgens [6]. Before assessing SFN responsiveness, we confirmed that these cells retained their characteristic identities by immunocytochemistry (ICC). SEB-1 cells expressed the sebocyte marker fatty acid synthase (FASN), whereas DPCs showed robust expression of the dermal papilla marker versican (VCAN), lymphoid enhancer binding factor 1 (LEF1), alkaline phosphatase (ALP), and the proliferating cell marker Ki67 (Fig. 4b) [25]. We also confirmed preserved androgen responsiveness in SEB-1 cells, as DHT treatment induced nuclear translocation of AR in vitro (Fig. 4c). We then treated SEB-1 cells and DPCs with SFN and observed strong upregulation of *AKR1Cs* in both cell types, while the induction patterns differed between them, with SFN-induced *AKR1C4* expression in SEB-1 cells and *AKR1C1–4* expression in DPCs attenuated by co-treatment with DHT (Fig. 4d-k). In SEB-1 cells, AKR1C1, particularly AKR1C4, showed the most prominent increase, whereas in DPCs, all AKR1Cs were elevated, but AKR1C1 and AKR1C3 were most strongly induced. Notably, AKR1C4 was detectable by qPCR even in steady-state SEB-1 cells and DPCs, despite being absent from many RNA-seq datasets. These findings demonstrate that SFN enhances AKR1C expression, including AKR1C4, in SG cells and DPCs, and suggest that AKR1C modulation may influence local DHT metabolism.

## Discussion

Systemic sex steroid levels decline with age, whereas clinical manifestations of androgen action remain highly tissue-specific, indicating a major contribution of local steroid metabolism in peripheral organs, including skin and its appendages [1]. In this study, we identified the scalp SG as a key, previously underappreciated, site of age-associated modulation of androgen metabolism in the human scalp. While 5αR activity in HFs is a well-established driver of androgen-dependent scalp disorders, including androgenetic alopecia (AGA), much less attention has been paid to the opposing inactivation pathways that convert DHT to weak metabolites [10, 11]. Our analysis shows that these reductive pathways, mediated by members of the AKR1C family, are robustly represented in human scalp SGs in youth and exhibit a progressive decline with age, particularly in males. This finding suggests that the aging scalp is characterized not only by active androgen synthesis but also by a diminishing capacity to buffer local steroid potency.

The detection of AKR1C1/2/3 proteins in scalp SGs is consistent with previous reports of steroid-metabolizing enzyme expression in human skin, suggesting that the skin, including its appendages, functions as an active steroidogenic and steroid-metabolizing organ [24, 26, 27]. In contrast, the presence of AKR1C4 protein in SGs and HFs represents, to our knowledge, a novel observation at the level of human scalp tissue. Although AKR1C4, historically considered liver specific [4, 28], possesses the highest 3α-HSD activity among the family members toward DHT and other 5α-reduced steroids, our IHC and qPCR results demonstrate its presence in the pilosebaceous unit. Previous transcriptomic datasets have rarely reported AKR1C4 in skin, which can be plausibly explained by multi-mapping of short reads among the highly homologous AKR1C genes, leading to underestimation of individual members [29]. The coordinated age-dependent decline of multiple AKR1Cs suggests that the aging scalp experiences a coordinated loss of DHT-inactivating capacity.

Functionally, this enzymatic decline is likely to weaken protection against excessive androgen exposure. AKR1C enzymes catalyze the reductive conversion of DHT to 3α/β-androstanediol, decreasing AR agonist potency by generating weaker metabolites [30, 31]. The high AKR1C expression observed in SGs from young male scalps may therefore act as a local buffer that limits DHT signaling in the perifollicular microenvironment, despite relatively high circulating androgens in early adulthood. Moreover, SG atrophy observed in HFs affected by scarring alopecia and seborrheic dermatitis suggests the critical role of SGs in HF maintenance [32, 33]. The marked sex difference in AKR1C expression observed in SGs represents a notable finding and may help explain why the male scalp, which is continuously exposed to high androgen levels and thus appears to rely heavily on the SG buffering system, is particularly vulnerable to the age-related loss of reductive capacity.

Our findings on AR localization further refine our understanding of androgen responsiveness in the human scalp. Classical models often focus on DPCs as primary androgen targets based on detection of AR and 5αR in balding scalp follicles [12]. However, in our study, AR immunoreactivity was consistently nuclear in SGs and a subset of sweat glands, but not detectable in DPCs or bulge regions using two independent antibodies. This result suggests that SGs represent a major androgen-responsive compartment in the human scalp. Given the anatomical and functional coupling between SGs and HFs, AR-dependent signaling within SGs may influence HF behavior through paracrine interactions. Moreover, in SEB-1 cells, although DHT stimulation confirmed AR responsiveness (Fig. 4c), the AR detected by the N-terminal antibody predominantly remained cytoplasmic in vitro. Together with the strong nuclear FL-AR signals observed specifically at the peripheral regions of SGs in vivo, these findings suggest that this compartment may function as a niche that supports the maintenance and nuclear activation of FL-AR.

In addition to FL-AR, several alternatively spliced AR isoforms have been described in normal tissues, including AR45, a truncated variant lacking most of the N-terminal transactivation domain, which has been reported in organs such as the heart and brain [34, 35]. AR45 has been proposed to act as a dominant-negative regulator of canonical AR signaling. In the present study, however, the cytoplasmic AR signal observed in the epidermal granular layer was detected using antibodies recognizing N-terminal epitopes, indicating that this signal is unlikely to represent AR45. Our findings are consistent with the expression of C-terminally truncated AR splice variants that retain the N-terminal domain, such as AR-V1, AR-V4, or AR-V6, which have been reported to localize predominantly in the cytoplasm and lack intrinsic transcriptional activity when expressed alone [36].

Previous studies have demonstrated divergent roles of AR signaling in cutaneous wound healing. While castration-mediated androgen deprivation and myeloid-specific AR knockout accelerate wound repair, keratinocyte-specific AR knockout paradoxically delays re-epithelialization, indicating a protective role of AR signaling in epidermal cells [37, 38]. These genetic studies assume that keratinocyte AR functions predominantly as the transcriptionally active FL-AR. In our immunohistochemical analyses, AR detected in the epidermal granular layer was predominantly cytoplasmic and was detected only with an antibody recognizing the N-terminal region of AR. In contrast, an antibody preferentially detecting FL-AR failed to detect AR in this compartment, supporting the possibility that keratinocytes express a C-terminally truncated, transcriptionally inactive AR splice variant rather than simple cytoplasmic retention of FL-AR. This raises the possibility that keratinocyte-specific AR deletion may eliminate such inhibitory AR splice variants, thereby altering the balance of androgen signaling during epidermal differentiation and barrier restoration. From a physiological perspective, expression of inhibitory AR splice variants in keratinocytes may suppress AR signaling in the epidermis while preserving androgen-dependent functions in SGs and HFs. This spatial segregation may be particularly important for maintaining epidermal homeostasis and preparation for tissue repair following injury.

The experimental data with SFN highlight the regulatory plasticity of AKR1C-mediated inactivation pathways. AKR1C genes are known targets of the transcription factor NRF2, and activation of this pathway can upregulate multiple AKR1C isoforms in various cell types. In our in vitro assays, SFN robustly induced AKR1C1/2/3 and AKR1C4 in SEB-1 sebocytes and DPCs, suggesting that local androgen-inactivating capacity may be pharmacologically enhanced. Notably, SFN has demonstrated favorable tolerability profiles in multiple human intervention studies without serious adverse events [39]. Together with prior evidence that skin and its appendages possess intrinsic steroidogenic and steroid-metabolizing activity [24, 26, 27], these findings raise the possibility that enhancing catabolic pathways within the scalp could reduce local androgen burden without globally suppressing androgen synthesis, potentially offering a safer, tissue-targeted alternative to systemic 5αR inhibition, which is associated with adverse effects in some individuals.

Recent studies have also demonstrated that glucocorticoid signaling contributes to the maintenance of HF stem cell quiescence [40]. Since both 5α/βRs and 3α-HSDs participate in the metabolism of androgens and glucocorticoids, our findings raise the possibility that SGs function as terminal regulatory sites for local steroid hormone activity in the human scalp. This concept expands the conventional view of SGs from passive lipid-secreting appendages to active endocrine-modulatory units. However, the present human tissue analyses demonstrate a correlative association and do not establish a direct causal relationship between age-associated AKR1C reduction and hair cycle abnormalities or AGA progression. Functional validation will require the development of experimental systems incorporating SG cells, including organoid and ex vivo tissue culture models, to enable direct manipulation of AKR1C activity and to assess its effects on HF maintenance and regeneration. Although comprehensive gene expression and functional datasets encompassing all relevant scalp compartments, including SGs, HFs, and subcutaneous tissues, remain scarce, future studies integrating transcriptomics, targeted steroid metabolomics, and functional assays will be essential for elucidating inter-tissue steroid dynamics and may help inform future therapeutic approaches for age-associated hair loss through modulation of local hormone metabolism.

## Conclusions

In summary, we identified scalp SGs as a previously underappreciated site of androgen inactivation mediated by AKR1Cs, and demonstrated that their expression, including that of AKR1C4, declines with age and shows sex-specific patterns. These findings suggest that age-associated loss of AKR1C activity may contribute to elevated local androgen signaling within the scalp microenvironment and the development of age-associated hair loss. Our results highlight the potential of targeting local steroid metabolism in SGs to HF homeostasis during aging, which may help inform future therapeutic approaches for age-associated hair loss.

## Supplementary Information

Supplementary Material 1: Figure S1. Full uncropped gels and blot images.

Supplementary Material 2: Figure S2. Validation of AR localization using an independent anti-AR antibody. High-magnification IHC images of human scalp tissues stained with a second AR antibody (AR (CST), Cell Signaling Technology, #5153), together with Ki67. Lower panels show enlarged views of representative regions with individual AR, Ki67, and DAPI channels and their merged images. Representative images show nuclear AR signals in SGs and sweat glands, consistent with the distribution observed in Fig. 3, whereas AR staining was minimal or undetectable in the epidermis and HF infundibulum. Occasional DAPI-negative red signals observed around the outer root sheath likely represent nonspecific staining of erythrocytes and were not considered as true AR-positive cells. Scale bars, 100 µm (upper panels) and 20 µm (lower panels).

Supplementary Material 3: Table S1. GEO data information.

Supplementary Material 4: Table S2. Antibodies.

Supplementary Material 5: Table S3. Primers used for qPCR analysis.

Supplementary Material 6: Table S4. Multiple linear regression of AKR1C1/2/3 expression in male sebaceous glands.

Supplementary Material 7: Table S5. Multiple linear regression of AKR1C4 expression in male sebaceous glands.

Supplementary Material 8: Table S6. Multiple linear regression of AKR1C4 expression in hair follicles.

## Data Availability

All data generated or analyzed during this study are included in this article. Further inquiries can be directed to the corresponding authors.

## Abbreviations

AGA: Androgenetic alopecia
DPCs: Dermal papilla cells
DHT: Dihydrotestosterone
SRD5A: Steroid 5 alpha-reductase
AKR1C: Aldo-keto reductase family 1 member C
HSD: Hydroxysteroid dehydrogenase
5αR: 5α-Reductase
SGs: Sebaceous glands
HFs: Hair follicles
HEK293T: Human embryonic kidney 293T
AR: Androgen receptor
NFE2L2: NFE2 like bZIP transcription factor 2
FASN: Fatty acid synthase
VCAN: Versican
LEF1: Lymphoid enhancer binding factor 1
SFN: Sulforaphane
qPCR: Quantitative polymerase chain reaction
