# Supplementary figures and images for: Age-related downregulation of dihydrotestosterone-inactivating enzymes in human scalp sebaceous glands
Source: Inflamm Regen. 2026 Apr 7;46:20. doi: 10.1186/s41232-026-00417-5 (PMC13126766; doi:10.1186/s41232-026-00417-5)

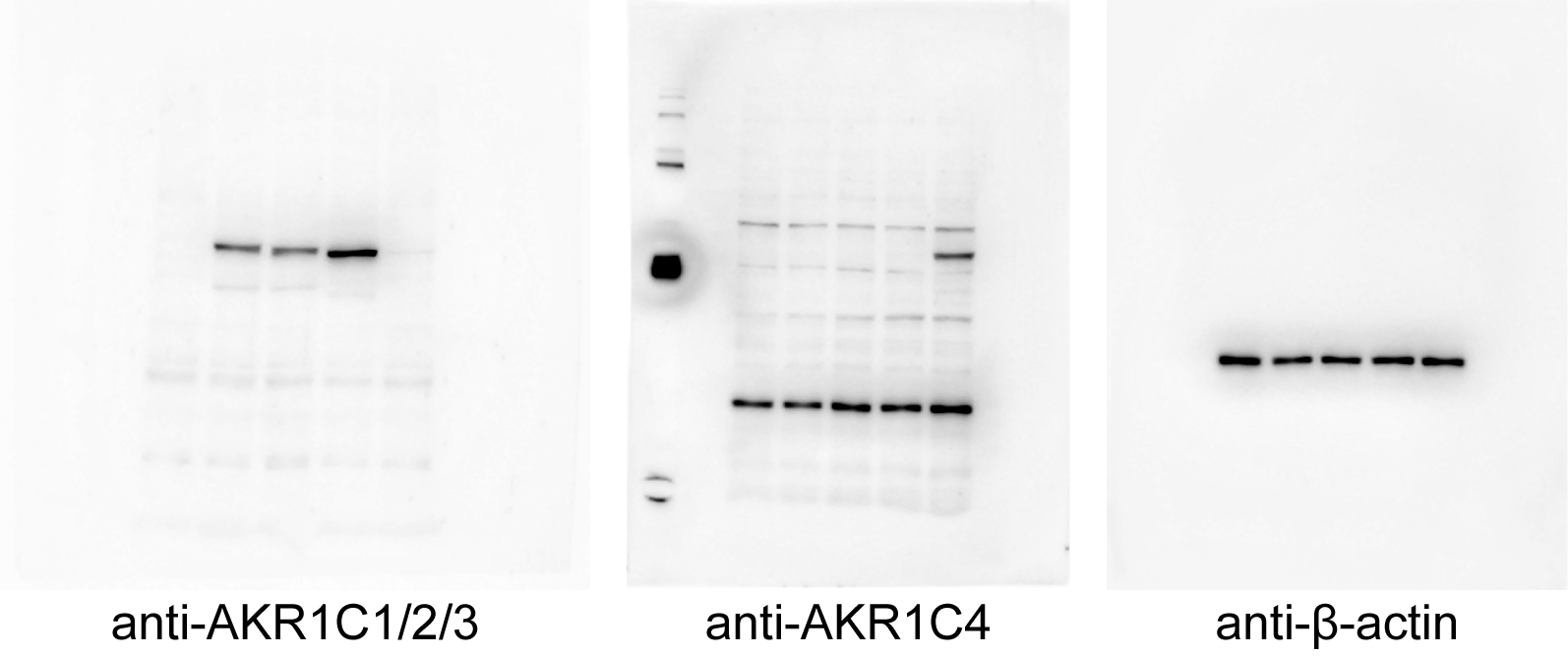

Supplement: Supplementary file 1 — Supplementary Material 1: Figure S1. Full uncropped gels and blot images. [file 41232_2026_417_MOESM1_ESM.tif]

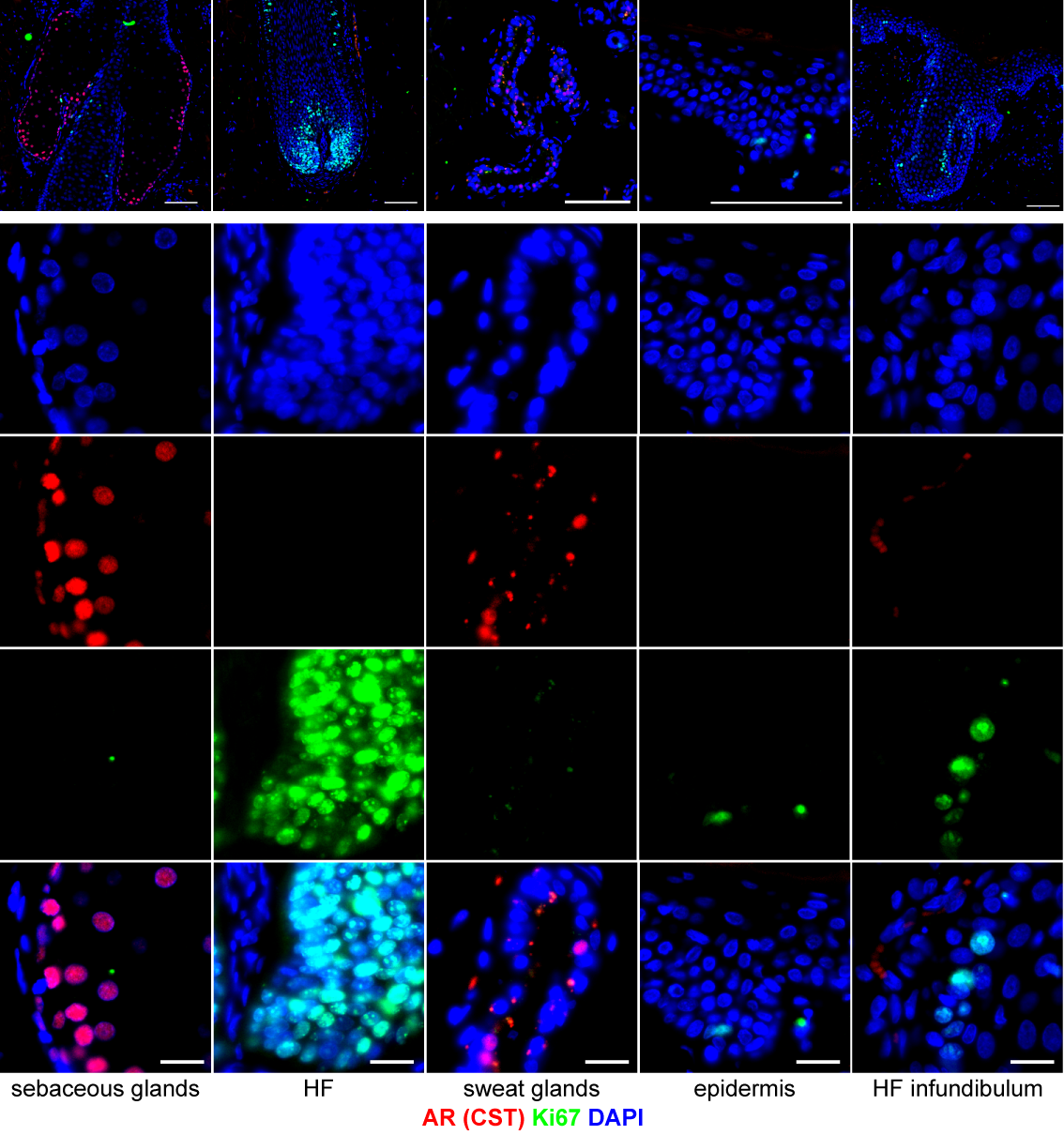

Supplement: Supplementary file 2 — Supplementary Material 2: Figure S2. Validation of AR localization using an independent anti-AR antibody. High-magnification IHC images of human scalp tissues stained with a second AR antibody (AR (CST), Cell Signaling Technology, #5153), together with Ki67. Lower panels show enlarged views of representative regions with individual AR, Ki67, and DAPI channels and their merged images. Representative images show nuclear AR signals in SGs and sweat glands, consistent with the distribution observed in Fig. 3, whereas AR staining was minimal or undetectable in the epidermis and HF infundibulum. Occasional DAPI-negative red signals observed around the outer root sheath likely represent nonspecific staining of erythrocytes and were not considered as true AR-positive cells. Scale bars, 100 µm (upper panels) and 20 µm (lower panels). [file 41232_2026_417_MOESM2_ESM.tif]
